# Feasibility, safety, and efficacy of circumferential spine stereotactic body radiotherapy

**DOI:** 10.3389/fonc.2022.912799

**Published:** 2022-11-23

**Authors:** Khaled Dibs, Joshua D. Palmer, Rahul N. Prasad, Alexander Olausson, Eric C. Bourekas, Daniel Boulter, Ahmet S. Ayan, Eric Cochran, William S. Marras, Prasath Mageswaran, Evan Thomas, John Grecula, Michael Guiou, Soheil Soghrati, Esmerina Tili, Raju R. Raval, Ehud Mendel, Thomas Scharschmidt, James B. Elder, Russell Lonser, Arnab Chakravarti, Dukagjin M. Blakaj

**Affiliations:** ^1^ Department of Radiation Oncology, The James Cancer Center at the Ohio State University Wexner Medical Center, Columbus, OH, United States; ^2^ Department of Radiology, The James Cancer Center at the Ohio State University Wexner Medical Center, Columbus, OH, United States; ^3^ Department of Biomedical Engineering, Spine Research Institute, The Ohio State University, Columbus, OH, United States; ^4^ Department of Radiation Oncology, Green Bay Oncology, Green Bay, WI, United States; ^5^ Department of Mechanical and Aerospace Engineering at the Ohio State University, Columbus, OH, United States; ^6^ Department of Anesthesiology, Ohio State College of Medicine, Columbus, OH, United States; ^7^ Department of Neurosurgery, School of Medicine, Yale University, New Haven, CT, United States; ^8^ Department of Orthopedic Surgery, The James Cancer Center at the Ohio State University Wexner Medical Center, Columbus, OH, United States; ^9^ Department of Neurosurgery, The James Cancer Center at the Ohio State University Wexner Medical Center, Columbus, OH, United States

**Keywords:** circumferential, epidural disease, local control, radiosurgery, spine metastases, stereotactic body radiation therapy, stereotactic radiotherapy, toxicity

## Abstract

**Background:**

With advances in systemic therapy translating to improved survival in metastatic malignancies, spine metastases have become an increasingly common source of morbidity. Achieving durable local control (LC) for patients with circumferential epidural disease can be particularly challenging. Circumferential stereotactic body radiotherapy (SBRT) may offer improved LC for circumferential vertebral and/or epidural metastatic spinal disease, but prospective (and retrospective) data are extremely limited. We sought to evaluate the feasibility, toxicity, and cancer control outcomes with this novel approach to circumferential spinal disease.

**Methods:**

We retrospectively identified all circumferential SBRT courses delivered between 2013 and 2019 at a tertiary care institution for post-operative or intact spine metastases. Radiotherapy was delivered to 14–27.5 Gy in one to five fractions. Feasibility was assessed by determining the proportion of plans for which ≥95% planning target volume (PTV) was coverable by ≥95% prescription dose. The primary endpoint was 1-year LC. Factors associated with increased likelihood of local failure (LF) were explored. Acute and chronic toxicity were assessed. Detailed dosimetric data were collected.

**Results:**

Fifty-eight patients receiving 64 circumferential SBRT courses were identified (median age 61, KPS ≥70, 57% men). With a median follow-up of 15 months, the 12-month local control was 85% (eight events). Five and three recurrences were in the epidural space and bone, respectively. On multivariate analysis, increased PTV and uncontrolled systemic disease were significantly associated with an increased likelihood of LF; ≥95% PTV was covered by ≥95% prescription dose in 94% of the cases. The rate of new or progressive vertebral compression fracture was 8%. There were no myelitis events or any grade 3+ acute or late toxicities.

**Conclusions:**

For patients with circumferential disease, circumferential spine SBRT is feasible and may offer excellent LC without significant toxicity. A prospective evaluation of this approach is warranted.

## Introduction

With recent advances in systemic therapy translating to improved survival for patients with metastatic malignancies, spine metastases have become an increasingly common source of morbidity for patients (1–3). Radiation therapy (RT) +/- surgery is typically offered for patients requiring palliation due to poorly controlled pain or progressive neurologic symptoms (3). Conventional external beam RT has traditionally been considered the standard of care for patients receiving palliative RT (3). However, the biologically effective dose (BED) delivered by conventional RT can be ineffective in some cases—particularly for patients with radioresistant histologies (4, 5).

Spine stereotactic body radiotherapy (SBRT), which offers the ability to safely deliver a higher BED to tumor through more precise treatment planning and delivery, has demonstrated improved local control (LC) in multiple prospective trials (6, 7). However, because adjacent normal tissues may also receive a higher BED, vertebral compression fracture (VCF) (8–10) and radiation-induced spinal cord injury (11) are potential late consequences of SBRT. In patients with circumferential or extensive epidural disease, consensus contouring guidelines recommend covering the affected spinal levels with circumferential RT delivered to all six vertebral compartments (12, 13). Because of the need for circumferential RT and the likelihood of abutment or near-abutment of critical neural structures by disease, conventional RT is frequently offered to these patients to minimize the risk of high-grade toxicity with SBRT. However, without the higher BED offered by SBRT, these patients are at risk for local failure (LF) in a particularly morbid location adjacent to the spinal cord.

Circumferential SBRT, if feasible, could improve the rates of LF in patients with circumferential or near-circumferential spinal metastases. Yet, to date, prospective and retrospective data evaluating the feasibility, toxicity, and cancer control outcomes with circumferential SBRT is extremely limited. Thus, we sought to evaluate the feasibility of this approach as well as assess the rates of LC and toxicity.

## Method

### Study design

We retrospectively identified patients with spine metastases treated with circumferential SBRT at a single tertiary care center between 2013 and 2019. Patients who underwent surgical resection prior to SBRT were included. RT courses delivered for cervical, thoracic, lumbar, or sacral metastases were all included. Patient characteristics were gathered including age, gender, and performance status as defined by the Karnofsky Performance Status (KPS). Disease characteristics including tumor histology, degree of control of systemic disease, site of spine metastases (e.g., cervical spine), spinal instability neoplastic score (SINS), and Bilsky grade were collected. If surgical intervention was offered, especially in patients with Bilsky grade 3, the type of surgery was documented. Information regarding SBRT courses was collected including the prescribed dose, fractionation, number of treated spinal levels, and the volume of the planning target volume (PTV). For the tumor, BED was calculated using an α/β ratio of 10; for normal tissue such as the spinal cord and cauda equina, an α/β ratio of 2 was used. From these BED values, dosimetric data were collected. These examined metrics included minimum and maximum doses to the PTV, the dose received by 95% of the PTV (D95%) and D90%, and max point doses to the spinal cord and cauda equina (where applicable). This study received approval from the institutional review board.

### Treatment

All patients completed magnetic resonance imaging (MRI) of the spine before surgery or spine SBRT. If patients received a surgical intervention, they completed post-operative MRI as well as a computed tomography (CT) myelogram on the day of simulation for radiation planning. CT simulation occurred in the supine position. With metallic implants, we performed the simulation with a metal artifact reduction (MAR) imaging protocol, and reconstruct to reduce the artifact. If the artifact persisted, we contour the darkening and streaking, overriding to tissue equivalent (40 HU). No intravenous contrast was administered. For the reproducible immobilization of the cervical spine, a thermoplastic mask was used. Otherwise, a stereotactic body frame and a vac loc bag were sufficient for the reproducible immobilization of patients undergoing treatment of the thoracic, lumbar, and/or sacral spine. Spine MRI sequences, including, but not limited to, axial and sagittal T1 postcontrast and T2, were fused to the CT simulation to assist with the accurate delineation of target volumes and organs at risk. For postoperative patients, CT myelogram and both pre- and post-operative MRI were fused.

The clinical target volume (CTV) included gross osseous and extraosseous disease plus bony anatomy at risk of harboring microscopic disease which consisted of all 6 compartments around the spinal cord as per consensus contouring guidelines for both postoperative and definitive spine SBRT (12, 13) [15, 16]. The PTV was equivalent to the CTV; no expansion was used. The spinal cord was contoured using the CT myelogram for post-operative patients or the MRI T2 axial series for nonoperative patients. A circumferential expansion of 2 mm was used to create a planning risk volume (PRV) avoidance structure. SBRT was prescribed to a dose of 14–27.5 Gy in one to five fractions. Plans were normalized with the goal that at least 95% of the PTV would receive a full prescription dose. Cord constraints were as per AAPM TG 101 and thus varied based upon chosen fractionation [17]. In our department, we applied the maximum point dose to the spinal cord PRV. In some rare circumstances, as per physician’s discretion, we used true spinal cord maximum dose if the GTV was undercovered. According to TG 101 (14), the BED2 max point doses for one, three, and five fractions were 112, 101.8, and 120 Gy, respectively, for the spinal cord and 144, 120, and 134.4 Gy, respectively, for the cauda equina. Thus, for the dose tolerance of the spinal cord and cauda equina, volume-dose constraints of 120 Gy and 144 Gy by BED2 were set for planning with no more than 0.35 cc allowed to exceed these constraints. Volumetric modulated arc therapy was predominately used for treatment planning, and the plans typically utilized multiple coplanar arcs. Daily treatment occurred on a linear accelerator with 6 *df* couch capability and daily cone beam CT (CBCT) to maximize setup reproducibility.

### Study endpoints

The primary study endpoint was the rate of LC at 1 year. LC was assessed *via* follow-up MRIs of the treated spine completed every 3 months post-SBRT. Patients additionally completed clinic appointments every 3 months which consisted of a detailed history and physical exam. Imaging concerns for radiographic evidence of progression were retrospectively reviewed for confirmation of the local LF by two independent neuroradiologists. Secondary endpoints included progression-free survival (PFS), overall survival (OS), and rates of acute and chronic toxicity. PFS was defined as the time from the start of SBRT to first progression, death, or last follow-up. OS was defined as the time from the start of SBRT to the time of death or last follow-up. Acute and chronic toxicity were defined as per the National Cancer Institute Common Terminology Criteria for Adverse Events (CTCAE) v5.0. The proportion of plans where 95% of the PTV was covered by at least 95% of the prescription dose was assessed to determine the feasibility of this treatment approach.

### Statistical analysis

Descriptive statistics were conducted with continuous variables described using medians and ranges. Discrete variables were described using frequency counts and proportions. Median follow-up was calculated using the reverse Kaplan–Meier (KM) method (15). KM curves were used to assess the endpoints of LC, OS, and PFS. Univariate analysis was performed using a proportional hazards model to assess for a significant relationship between clinically relevant variables and increased likelihood of LF. For the analysis, continuous variables were dichotomized by the median. Cox proportional hazards modeling multivariate analyses were performed for LC. All statistical analysis was performed using SPSS Statistics version 27 (Armonk, NY).

## Results

### Patient and treatment characteristics

We identified 58 patients with 64 circumferential spine SBRT courses with a median follow-up of 15 months (range: 1–63 months) (**Table 1**). The median patient age at the time of treatment was 61 (range: 25–79), 57% of patients were men, and 88% of patients had a KPS ≥70; 39% of courses were associated with vertebrectomy, 21% with laminectomy, and 40% were not accompanied by surgery. The most prescribed regimen was 27 Gy in three fractions. SBRT to 3–7 vertebral levels was most common (46%) followed by 1 VB or 2 VB (27% for each). The most common treated histologies were renal cell carcinoma (31%), non-small cell lung cancer (12%), and soft tissue sarcoma (12%). The most treated portion of the spine was the thoracic spine (59%). Systemic disease burden was typically stable (75%). A SINS score of 8 or higher was most common (52%).

**Table 1:** Patient demographic and treatment characteristics.

Variable	Number
Number of Patients	58
Number of Courses	64
Median Follow-up (months) (Range)	15 (1–63)
Median Age (yr.) (Range)	61 (25–79)
Gender	
Male	37 (57%)
Female	27 (43%)
Karnofsky Performance Status	
≥70%	56 (88%)
<70%	8 (12%)
Surgery	
Vertebrectomy	25 (39%)
Laminectomy	13 (21%)
None	26 (40%)
Median Prescription Dose (Gy) (Range)	27.0 (14.0–27.5)
Median Prescription Dose BED* (Gy) (Range)	51.3 (33.6–51.3)
Median PTV Minimum BED* (Gy) (Range)	29.3 (10.8–44.0)
Median PTV Mean BED*(Gy) (Range)	54.1 (36.7–63.0)
Median PTV D95% BED* (Gy) (Range)	51.3 (26.6–52.0)
Median PTV D90% BED* (Gy) (Range)	52.0 (34.8–54.7)
Median Spinal Cord max dose BED** (Gy) (Range)	59.0 (15.0–114.0)
Median Cauda equina max dose BED** (Gy) (Range)	68.0 (7.5–136.0)
Median Spinal cord max dose (Gy)	
1 fraction (Range)	10 (8.8-11)
3 fractions (Range)	16.5 (7.2-22.77)
5 fractions (Range)	19 (5-29)
PTV coverage	
≥ 95% PTV covered by ≥ 95% Prescription Dose	60 (94%)
< 95% PTV covered by ≥ 95% Prescription Dose	4 (6%)
Fractionation	
Single Fraction	8 (12%)
Multi Fraction	56 (88%)
Median Treated Volume (cc) (Range)	132.0 (24.0–670.0)
Number of Treated Spinal Levels	
1 level	17 (27%)
2 levels	17 (27%)
3-7 levels	30 (46%)
Histopathology	
Renal Cell Carcinoma	20 (31%)
Sarcoma	8 (13%)
Non-small Cell Lung Cancer	8 (13%)
Breast	7 (11%)
Thyroid	5 (9%)
Prostate	4 (6%)
Gastrointestinal Adenocarcinoma	3 (5%)
Melanoma	3 (5%)
Head and Neck	2 (3%)
Neuroendocrine Tumor	1 (1%)
Metastatic Pituitary	1 (1%)
Metastatic Paraganglioma	1 (1%)
Bladder	1 (1%)
Vertebral site	
Cervical	6 (9%)
Cervical-Thoracic	1 (2%)
Thoracic	39 (59%)
Lumbar	10 (17%)
Thoracic-Lumbar	4 (6%)
Sacral	4 (7%)
Systemic disease status	
Stable	48 (75%)
Progression	16 (25%)
Spinal Instability Neoplastic Score Criteria	
< 7	12 (19%)
7	19 (29%)
> 7	33 (52%)
Bilsky grade	
1a/b	23 (36%)
1c	8 (13%)
2	18 (28%)
3	15 (23%)

PTV, planning target volume; (*)BED10; (**)BED2.

### Local control and survival outcomes

**Figure 1:**
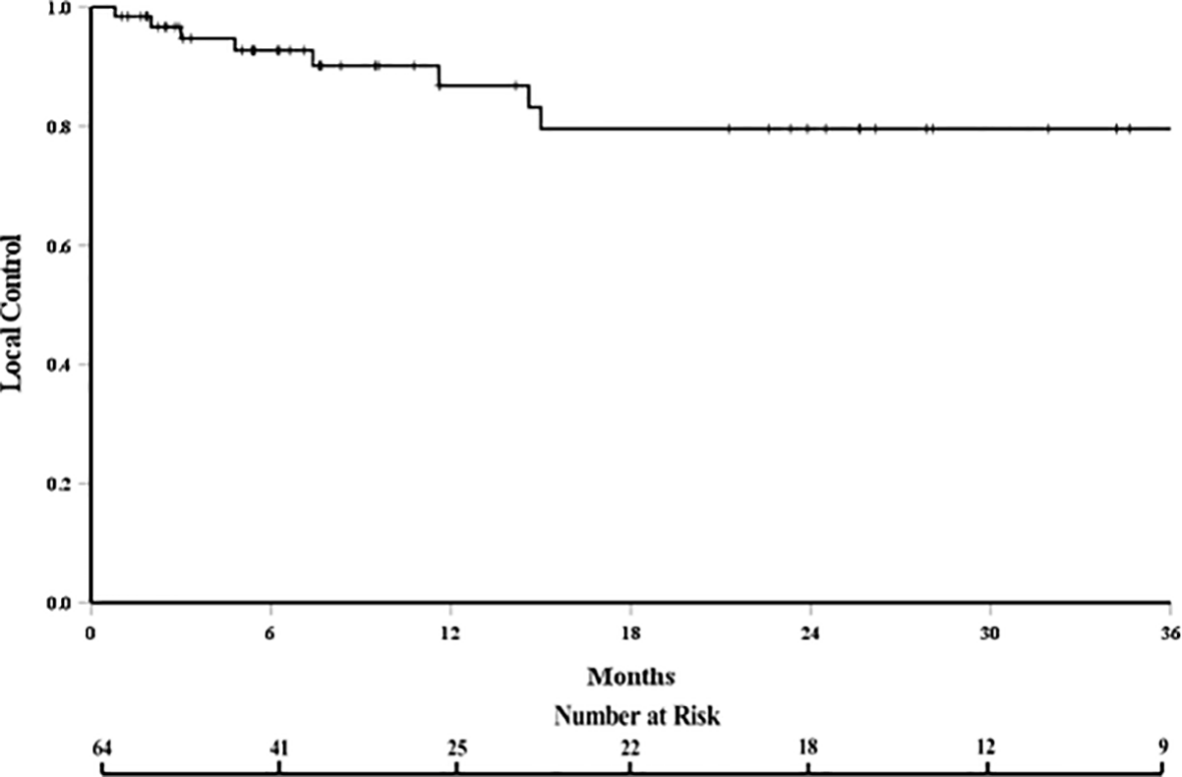
Local control curve.

**Figure 2:**
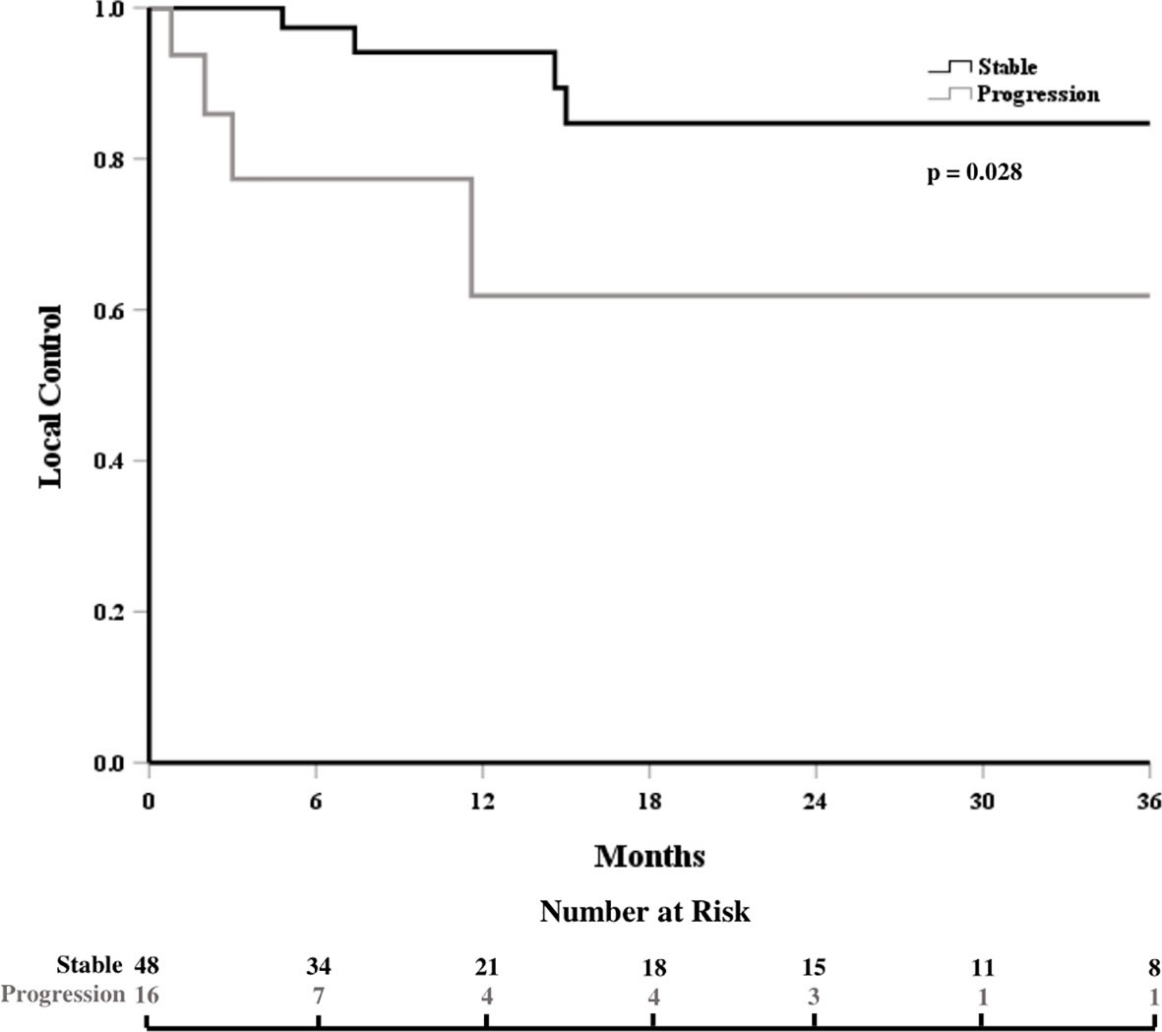
Local control, stratified by degree of systemic disease control at the time of treatment.

**Figure 3:**
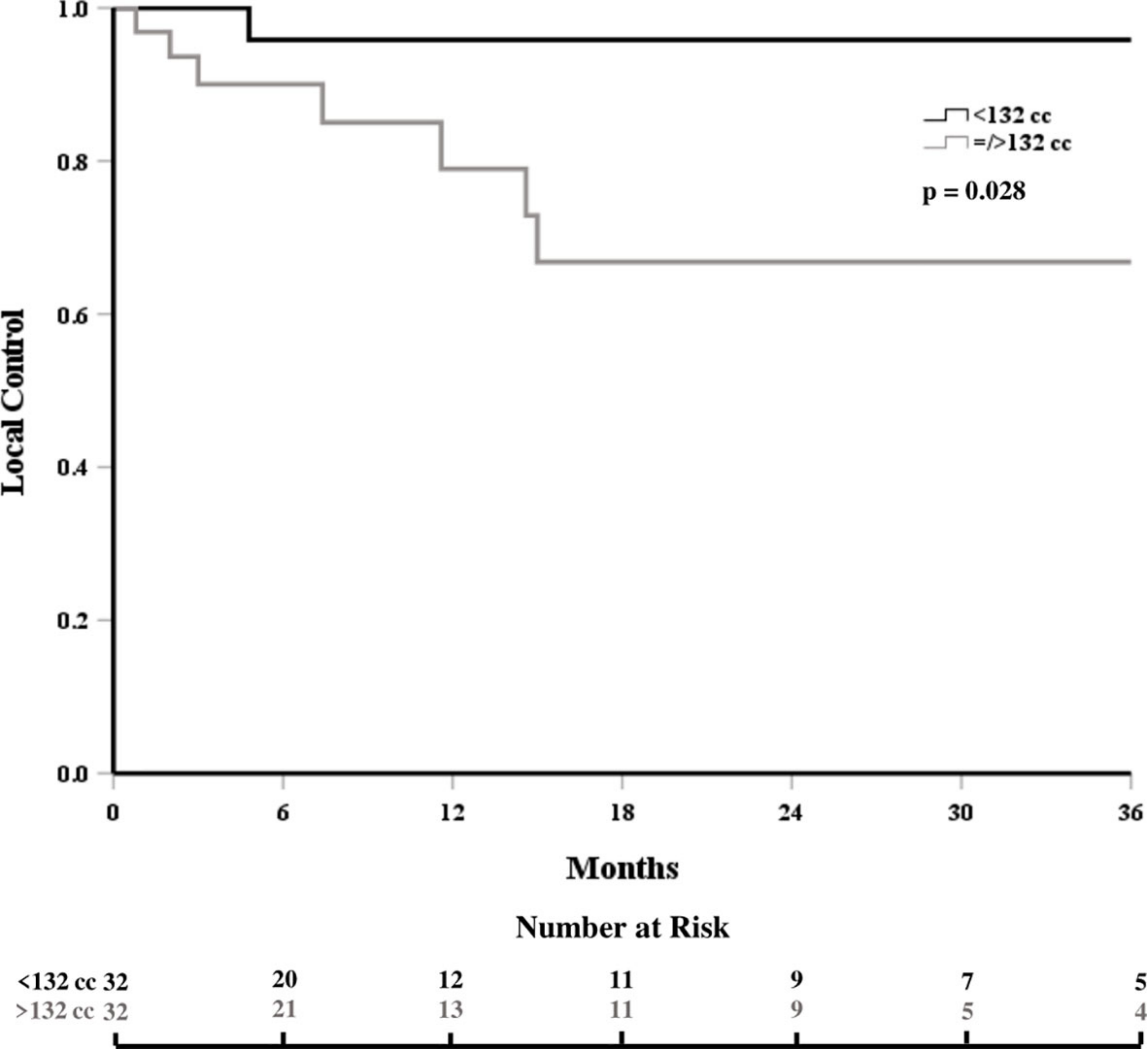
Local control, stratified by volume of treated disease.

The local control at 6, 12, and 18 months was 93%, 85%, and 80%, respectively (**Figure 1**). In patients who experienced LF, median time to LF was 8 months (range: 0.8–61). On univariate analysis, progressive systemic disease at the time of SBRT (1-year LC 94% vs. 62% for stable disease, p = 0.028, **Figure 2**) and a PTV greater than or equal to the median of 132 cc (1-year LC 96% vs. 79%, p = 0.042, **Figure 3**) were significantly associated with an increased likelihood of LF (**Table 2**). These variables remained significant on multivariate analysis (**Table 3**). No other pertinent demographic, disease, treatment, or dosimetric characteristics were significantly associated with an increased likelihood of LF on univariate or multivariate analyses. Of the eight LF events, the majority (five) occurred in the epidural space (**Table 4** *)*. Two additional LF events involved disease progression in the vertebral body, and the last LF involved a progression in the vertebral body with an extension to the right lateral compartment. Of the five patients with epidural recurrences, two elected for the hospice or died shortly after recurrence due to systemic progression, one was managed conservatively per patient preference due to the absence of cord compression, one required surgical resection, and one received reirradiation. The 1-year OS and PFS for this cohort were 60% and 56%, respectively.

**Table 2:** Univariate analysis examining for variables associated with increased likelihood of local failure using a proportional hazards model.

Variable	1-year Local Control	p value
Age		0.491
≤60	92%	
>60	81%	
Gender		0.718
Male	89%	
Female	84%	
Surgery vs no surgery		0.234
Vertebrectomy	76%	
Laminectomy	90%	
Definitive	96%	
Radiotherapy dose (BED)		0.652
≥51.3Gy	80%	
<51.3Gy	90%	
PTV minimum dose (BED)		0.486
≥29.2Gy	83%	
<29.2Gy	81%	
PTV mean dose (BED)		0.550
≥55.23Gy	90%	
<55.23Gy	74%	
D90% (BED)		0.604
≥52Gy	85%	
<52Gy	90%	
D95% (BED)		0.262
≥51.3Gy	83%	
<51.3Gy	92%	
PTV coverage		0.445
≥95%	86%	
<95%	100%	
Number of fractions		0.293
1	88%	
3	83%	
5	100%	
Treated volume		**0.042**
<132cc	96%	
≥132cc	79%	
Systemic disease status		**0.028**
Stable	94%	
Uncontrolled	62%	
SINS		0.44
≤7	96%	
>7	77%	
Bilsky		0.412
Grade 1a-c	96%	
Grade 2-3	76%	

BED, biologically effective dose; PTV, planning target volume; SINS, spinal instability neoplastic score. The bold values: statistically significant values.

**Table 3:** Multivariate analysis examining for variables associated with increased likelihood of local failure in patients with ≥3 contiguous treated levels using a proportional hazards model.

Variable	Hazard Ratio	95% Confidence Interval	p-value
Age	0.993	0.089–1.107	0.894
Gender	0.884	0.088–8.909	0.917
Surgery vs Definitive	8.652	0.634–118.000	0.106
Prescribed dose (BED)	1.372	0.269–7.000	0.704
Minimum dose (BED)	0.87	0.689–1.098	0.24
Mean dose (BED)	1.816	0.411–8.000	0.431
D90% (BED)	0.186	0.005–6.892	0.361
D95% (BED)	3.653	0.313–42.600	0.302
Treatment volume	1.011	1.001–1.021	**0.025**
Histopathology	0.85	0.600–1.200	0.395
SINS	0.543	0.207–1.430	0.216
Bilsky	0.022	0.001–3.600	0.143
Systemic disease status	27	1.700–428.000	**0.019**

BED, biologically effective dose. The bold values: statistically significant values.

**Table 4:** Summary of patients who developed recurrent epidural disease.

Case	Histopathology	Site	Prescribed dose	Minimum dose (%)	Mean dose (%)	Max dose (%)	Time to recurrence (months)	Max distance between the 95% isodose line and the inner target (mm)
1	NSCLC	Thoracic	27Gy/3fx	51	104	116	5	5
2	Prostate	Thoracic	27Gy/3fx	72	105	117	6	2
3	RCC	Thoracic	27Gy/3fx	76	104	111	12	4
4	Cholangiocarcinoma	Lumbar	18Gy/1fx	85	103	111	1	3
5	Colorectal	Lumbar	27Gy/3fx	51	105	113	14	6

NSCLC, non-small cell lung cancer; RCC, renal cell carcinoma; Gy, Gray; Fx, fraction.

### Feasibility of treatment delivery and dosimetry

**Figure 4:**
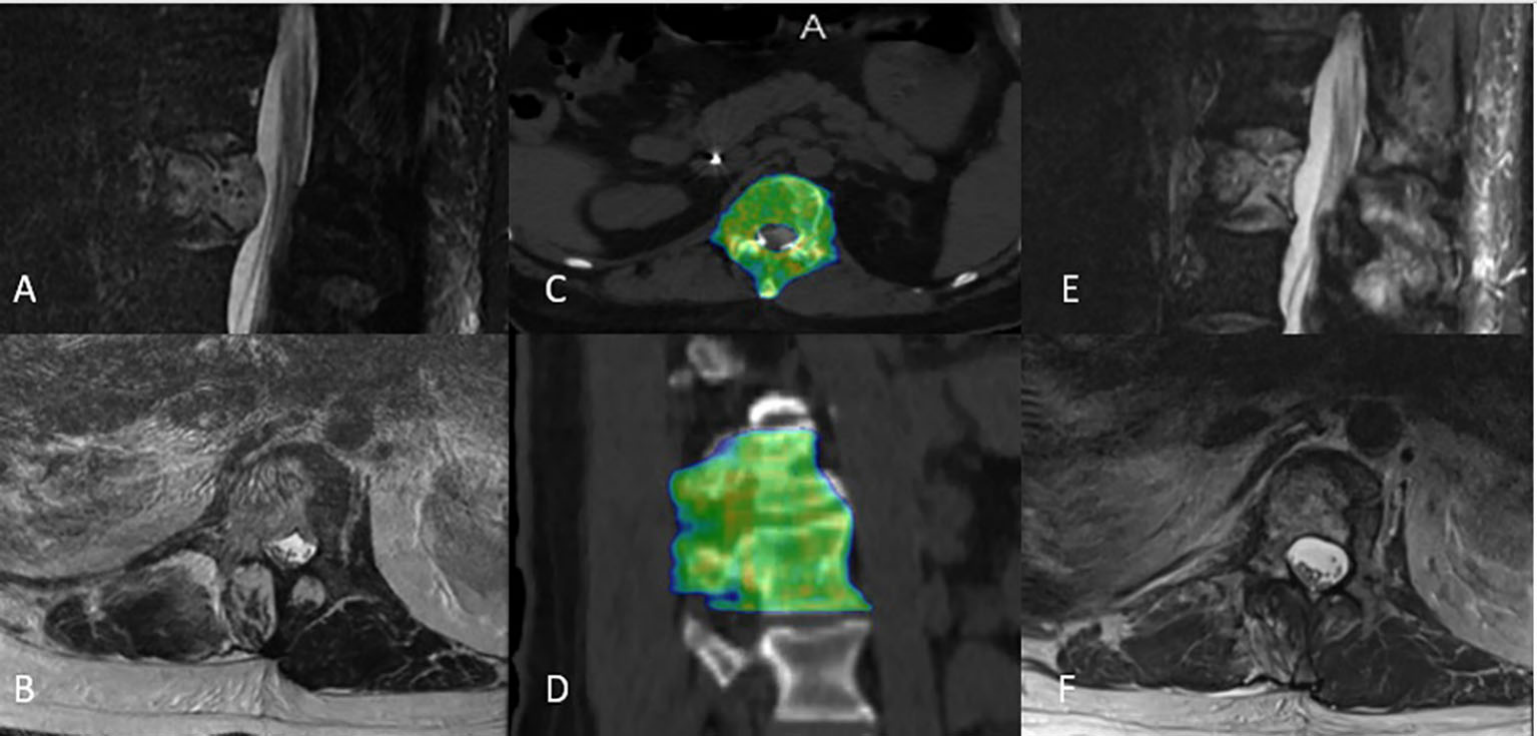
Representative circumferential stereotactic body radiation therapy plan from a patient treated to T12-L1 with **(A, B)** T2-weighted sagittal and axial MRI showing spinal canal narrowing due to extensive metastatic disease involving the vertebral body, right lateral compartment, and posterior compartment. **(C, D)** He was treated to 27 Gy in three fractions. **(E, F)** Surveillance T2-weighted sagittal and axial MRI showed with disease regression 4 months post-treatment.

The median PTV was 132 cc (range: 24–670 cc). The median prescribed dose to the tumor in BED was 51.3 Gy (range: 33.6–51.3). The median minimum dose to the PTV in BED was 29.3 Gy (range: 10.8–44.0). The median mean dose to PTV in BED was 54.1 Gy (range: 36.7–63.0). The median D95% in BED was 51.3 Gy (range: 26.6–52.0), and the median D90% in BED was 52.0 Gy (range: 34.8–54.7). The median max spinal cord and cauda point doses in BED were 59 Gy (range: 15.0–114.0) and 68 Gy (range: 7.5–136 Gy), respectively. In 94% of the cases, a coverage of at least 95% of the PTV with 95% of the prescription dose was achievable. Most of the patients received multifractionated radiotherapy (88%), and we found that patients with a higher number of treated levels tend to have multiple fractions, p = 0.023. **Figure 4** illustrates such a case and documents an example of disease regression after a successful course of circumferential SBRT. In this cohort, just one patient who received SBRT in three fractions exceeded the cutoff point of the spinal cord and cauda constraints; in this case, the actual max dose was 22.77 Gy.

### Acute and chronic toxicity

Around 51 and 35 cases were available for toxicity analysis at 6 and 12 months, respectively. Acute toxicity was noted in 23% of SBRT courses. Grade 1–2 fatigue (20%) was most common. Additional acute events included grade 1 shortness of breath, grade 1 skin reaction, and one pain flare episode treated with dexamethasone. No high-grade acute events were noted. No patients developed spinal cord myelopathy. Of the 26 definitive SBRT courses, two developed progressive VCF (8%), 16 patients had no VCF and did not develop VCF, and 10 had stable VCF during the follow-up period. No high-grade late toxicities were noted.

## Discussion

This study represents perhaps the first analyses to demonstrate that circumferential spine SBRT for extensive epidural and/or vertebral body disease is feasible, safe, and effective. From a feasibility standpoint, 94% of cases resulted in a coverage of at least 95% of the PTV with 95% of the prescription dose and just one barely exceeded established spinal cord and cauda dose constraints. No spinal cord myelopathy or other high-grade acute or chronic toxicities were observed. Rates of new or progressive VCF were extremely low. While spine SBRT is associated with an increased risk of vertebral compression fracture at rates of roughly 10%–15% (8–10), in our cohort, we observed just an 8% rate of progressive VCF with circumferential SBRT in nonoperative patients. Regarding effectiveness, a 1-year LC rate of 85% was excellent given the extent of disease in these patients necessitating circumferential therapy. This LC rate is comparable to other published cohorts of patients receiving non-circumferential spine SBRT [6, 21].

These findings have strong clinical relevance, because local therapy for spinal metastases presenting with circumferential or near-circumferential disease is uniquely challenging, and circumferential SBRT is an emerging option that may overcome these obstacles. Per consensus guidelines, patients who present with metastatic spine disease involving all six compartments of the vertebra or with near-circumferential/extensive epidural disease around the spinal cord or cauda equina require circumferential RT (12, 13). While historically this would frequently be delivered using conventional palliative RT, the BED achievable with conventional RT can be ineffective—particularly for patients with radioresistant disease processes—resulting in critical LF events (4). LF in patients with circumferential epidural disease can be particularly morbid due to the risk of cord compromise. Thus, if circumferential SBRT offers the ability to safely deliver a higher BED to tumor through more precise treatment planning and delivery, it may be critical to improving LC and decreasing the morbidity associated with recurrent disease (5). This issue will likely become increasingly important moving forward. Patients with spine metastases, some of which will present with circumferential disease, may present with increasing frequency as survival in the metastatic setting continues to improve with advances in systemic therapy (1–3).

Notably, in our cohort, progressive systemic disease and larger PTV volumes were associated with inferior LC after circumferential SBRT. Progressive systemic disease at the time of SBRT may portend a more treatment-resistant disease process. Consistent with a more extensive local disease burden, a larger PTV is an unsurprising risk factor for local progression after therapy. The majority of our LF events were in the epidural space (63%) which was consistent with a phase I/II study showing that 47% of recurrences occur in the epidural space (16). Given the extensive epidural disease in our cohort, our LF pattern was not surprising.

Some limitations of this approach and this analysis warrant mention. At our institution, we used a 0-mm expansion from CTV to PTV and a 2-mm PRV expansion around the spinal cord/cauda equina for both post-operative and definitive spine SBRT patients. However, a PTV expansion may be required in other practice settings depending upon institutional comfort with setup reproducibility. Ideal candidates for circumferential SBRT are likely well performing patients being considered for tumor resection who have excellent systemic therapy options. Otherwise, the potential benefit in improved LC with this technique, with respect to conventional RT, may not be meaningful. Additional limitations primarily stem from the study’s retrospective nature, including possible patient selection biases.

In summary, in appropriately selected patients, circumferential spine SBRT is feasible and may offer excellent rates of LC with minimal toxicity. Thus, the benefits of spine SBRT may be extendable to patients with circumferential epidural or vertebral disease who historically have been considered more suitable for conventional EBRT. Prospective confirmation of the benefits and toxicity of this approach is warranted.

## Data availability statement

The raw data supporting the conclusions of this article will be made available by the authors, without undue reservation.

## Ethics statement

The studies involving human participants were reviewed and approved by The Ohio State University Institutional Review Board. Written informed consent for participation was not required for this study in accordance with the national legislation and the institutional requirements.

## Author contributions

KD and JP – first authors; RP, AO, EB, DB, AA, EC, WM, PM, ET, JG, MG, SS, EMT, RR, EM, TS, JE, RL, and AC – contributors; DMB – senior author. All authors contributed to the article and approved the submitted version.

## Conflict of interest

The authors declare that the research was conducted in the absence of any commercial or financial relationships that could be construed as a potential conflict of interest.

## Publisher’s note

All claims expressed in this article are solely those of the authors and do not necessarily represent those of their affiliated organizations, or those of the publisher, the editors and the reviewers. Any product that may be evaluated in this article, or claim that may be made by its manufacturer, is not guaranteed or endorsed by the publisher.
